# Supplementary material for: Interactions between Cyanobacteria and Methane Processing Microbes Mitigate Methane Emissions from Rice Soils
Source: Microorganisms. 2023 Nov 21;11(12):2830. doi: 10.3390/microorganisms11122830 (PMC10745823; doi:10.3390/microorganisms11122830)
Supplement: Supplementary file 1 [file microorganisms-11-02830-s001.zip › microorganisms-2691760-supplementary.pdf]

Supplementary Material

Interactions between cyanobacteria and methane processing microbes mitigate methane emissions from rice soils

Pérez, G*., Krause, S. M.B., Bodelier, P.L.E., Meima-Franke, M., Pitombo, L., and Irisarri, P.

***g.perez@nioo.knaw.nl/gperez@fagro.du.uy**

Supplementary Figure S1. Experimental setup. Each microcosm contained sieved soil from a temperate rice paddy. Two cyanobacterial isolates, belonging to the genus *Nostoc* sp. and *Calothrix* sp. and isolated from the same rice paddy, were added on day 0. At each sampling date, after collecting the CH_4 samples, microcosms were destructed to collect soil samples for nucleic acid extraction and physicochemical determinations

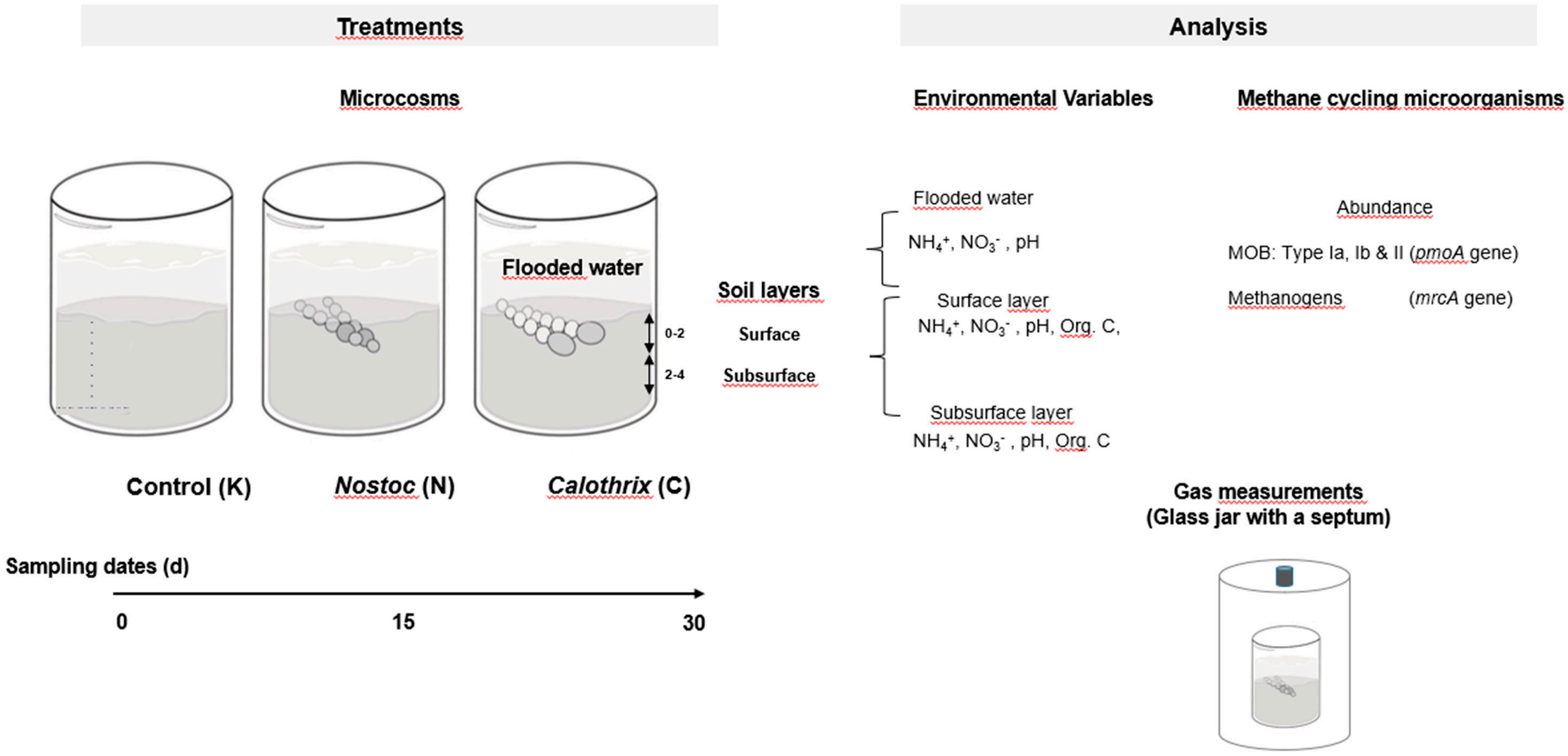

Supplementary Figure S2. Overview of the abundance and activity of MOB subgroups from both soil layers on day 0.

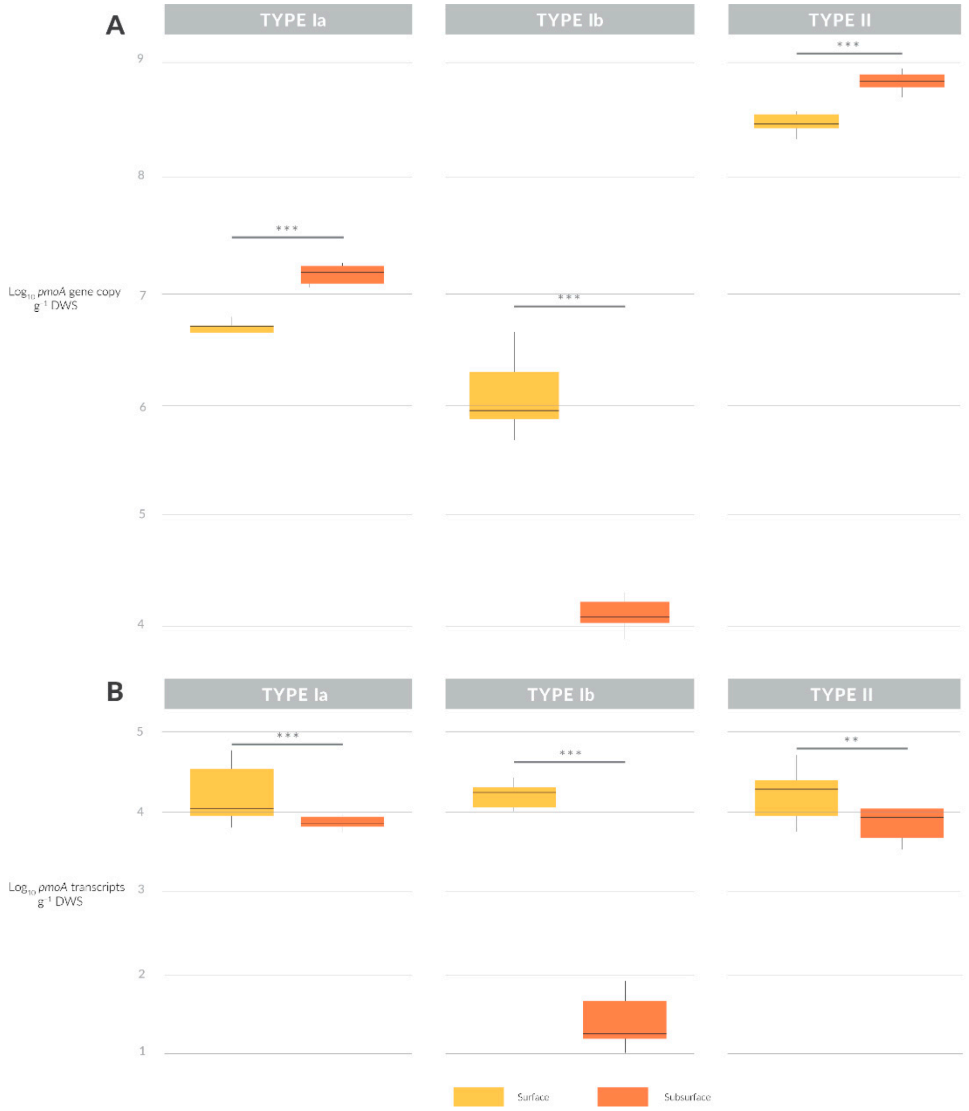

Supplementary Table S1.

Primers and PCR conditions used to amplify fragments of functional marker genes *pmoA* and *mrcA* by qPCR.

Gene	Bacterial group	Primer sets	PCR conditions	PCR product length (bp)	References
mrcA	methanogens	mlas/ mcrA-rev	95 °C/3min, 40 cycles (95°C/10sec, 60°C/10sec, 72°C/25sec), 65 to 95°C (+1°C/sec) for denaturation curve.	645	(Steinberg & Regan, 2008)
pmoA	MOB subgroup Ia	A189/ Mb601-rev	95 °C/3min, 45 cycles (95°C/10sec, 60°C/10sec, 72°C/25sec, 82°C/8sec), 70 to 99°C (+1°C/sec) for denaturation curve.	588	(Kolb *et al.*, 2003)
pmoA	MOB subgroup Ib	A189/Mc468-rev	95 °C/3min, 45 cycles (95°C/10sec, 64°C/10sec, 72°C/25sec, 82°C/8sec), 70 to 99°C (+1°C/sec) for denaturation curve.	455	(Kolb *et al.*, 2003)
pmoA	MOB subgroup II	I223- II646-rev	95 °C/3min, 45 cycles (95°C/10sec, 60°C/10sec, 72°C/25sec, 87°C/8sec), 70 to 99°C (+1°C/sec) for denaturation curve.	599	(Kolb *et al.*, 2003)

Steinberg, L.M., Regan, J.M., 2008. Phylogenetic comparison of the methanogenic communities from an acidic, oligotrophic fen and an anaerobic digester treating municipal wastewater sludge. Applied and environmental microbiology 74(21), 6663-6671.

Kolb, S., C. Knief, S. Stubner, and R. Conrad. 2003. Quantitative detection of methanotrophs in soil by novel *pmoA*- targeted real-time PCR assays. Applied and Environmental Microbiology 69:2423-2429.

Supplementary Table S2. Output of the mixed effect models (1-3) on MOB overall *pmoA* gene copies/transcripts and methanogens (*mrcA* gene copies). *p*-values of fixed effects from the Type III analysis of variance with Kenward-Roger's method.

Fixed effect	DF	F value	*p* value
		Type Ia *pmoA* gene copies	
Treatment	2	2.14	0.12
Incubation_period	2	1.64	0.20
Soil_layer	1	6.31	**0.01**
		Type Ib *pmoA* gene copies	
Treatment	2	1.34	0.27
Incubation_period	2	0.13	0.13
Soil_layer	1	69.65	**<0.01**
		Type II *pmoA* gene copies	
Treatment	2	0.86	0.42
Incubation_period	2	1.90	0.15
Soil_layer	1	7.97	**<0.01**
		Type Ia *pmoA* transcripts	
Treatment	2	0.48	0.61
Incubation_period	2	1.34	0.27
Soil_layer	1	24.41	**<0.01**
		Type Ib *pmoA* transcripts	
Treatment	2	5.29	**<0.01**
Incubation_period	2	0.01	0.92
Soil_layer	1	1284.9	**<0.01**
		Type II *pmoA* transcripts	
Treatment	2	2.51	0.09
Incubation_period	2	0.23	0.79
Soil_layer	1	12.01	**<0.01**
		mrcA gene copies	
Treatment	2	6.45	**<0.01**
Incubation_period	2	0.83	0.36
Soil_layer	1	1457.9	**<0.01**

Supplementary Table S3. Linear model output to explain the influence of inoculation, incubation period and their interaction on Type Ia *pmoA* gene copies in the surface soil layer on day 30. Significant differences were found when *p* values ≤ 0.05.

Fixed effect	DF	F value	*p* value
		Surface soil layer	
Treatment	2	2.63	0.09
Incubation_period	2	0.96	0.40
Treatment:Incubation_period	4	7.81	**<0.01**
		Subsurface soil layer	
Treatment	2	1.74	0.20
Incubation_period	2	1.75	0.20
Treatment:Incubation_period	4	0.72	0.59

Supplementary Table S4. Changes in water-column properties during the experiment. Values represent mean ± standard deviation. Units of ammonium and nitrate are mg N. kg^{-1} mL. Different lowercase letters on the same row show significant differences among days ($p \leq 0.05$).

Treatment	Variable	Water column Incubation period (d)		
		0	15	30
Control	pH	6.33 ± 0.13a	7.02 ± 0.84b	8.14 ± 0.21b
	NH_4^+	0.48 ± 0.68a	0.14 ± 0.03a	0.52 ± 0.41b
	NO_3^-	0.07 ± 0.10b	0.12 ± 0.03a	0.43 ± 0.03b
Nostoc	pH	6.39 ± 0.30a	7.68 ± 0.14b	8.09 ± 0.05b
	NH_4^+	0.50 ± 0.26a	0.26 ± 0.10a	0.10 ± 0.04b
	NO_3^-	0.04 ± 0.05b	0.09 ± 0.02a	0.39 ± 0.07b
Calothrix	pH	6.49 ± 0.25a	8.80 ± 0.12b	8.16 ± 0.03b
	NH_4^+	0.29 ± 0.40a	0.11 ± 0.02a	0.14 ± 0.02b
	NO_3^-	0.04 ± 0.06b	0.09 ± 0.02a	0.39 ± 0.07b

Supplementary Table S5. Linear model output to explain the influence of inoculation, incubation period and their interaction on the physicochemical variables in the studied compartments. Significant differences were found when *p* values ≤ 0.05. (-) means not determined.

		Compartments					
		Water-column		Surface soil layer		Subsurface soil layer	
		F	*p*	F	*p*	F	*p*
Variable	Term included						
pH	IP	80.1	< 0.01	7.39	< 0.01	8.03	< 0.01
	T	0.50	0.60	5.07	< 0.01	0.92	0.41
	IPxT	67.91	< 0.01	2.03	0.15	6.62	< 0.01
NH_4^+	IP	17.31	< 0.01	2.124	0.14	0.17	0.84
	T	0.01	0.99	0.01	0.99	1.69	0.21
	IPxT	3.31	0.02	0.72	0.68	0.43	0.88
NO_3^-	IP	33.80	< 0.01	11.14	< 0.01	10.43	< 0.01
	T	0.06	0.94	0.74	0.48	1.60	0.22
	IPxT	7.18	< 0.01	3.60	0.01	3.95	< 0.01
OC	IP	-	-	2.18	0.13	6.42	< 0.01
	T	-	-	8.61	0.001	4.81	0.03
	IPxT	-	-	10.29	< 0.01	8.20	< 0.01
